# Enhanced Production, Cloning, and Expression of a Xylanase Gene from Endophytic Fungal Strain *Trichoderma harzianum* kj831197.1: Unveiling the In Vitro Anti-Fungal Activity against Phytopathogenic Fungi

**DOI:** 10.3390/jof8050447

**Published:** 2022-04-25

**Authors:** Sawsan Abd Ellatif, Elsayed S. Abdel Razik, Ameena A. AL-surhanee, Faisal Al-Sarraj, Ghadir E. Daigham, Amira Y. Mahfouz

**Affiliations:** 1 Bioprocess Development Department, Genetic Engineering and Biotechnology Research Institute (GEBRI), City for Scientific Research and Technology Applications, New Borg El-Arab 21934, Egypt; 2 Plant Protection and Biomolecular Diagnosis Department, Arid Lands Cultivation Research Institute, City for Scientific Research and Technology Applications, New Borg El-Arab 21934, Egypt; eshabaan@srtacity.sci.eg; 3 Biology Department, College of Science, Jouf University, Sakaka 2014, Saudi Arabia; amaserhani@ju.edu.sa; 4 Department of Biological Sciences, Faculty of Sciences, King Abdulaziz University, Jeddah 21589, Saudi Arabia; falsaraj@kau.edu.sa; 5 Botany and Microbiology Department, Faculty of Science, Al-Azhar University (Girls Branch), Cairo 11435, Egypt; ghadirdaigham@azhar.edu.eg (G.E.D.); amira.mohamed@azhar.edu.eg (A.Y.M.)

**Keywords:** xylanase, submerged fermentation, cloning, *Trichoderma harzianum*, *E. coli*, phytopathogenic fungi

## Abstract

*Trichoderma* sp. is extensively applied as a beneficial fungus for the management of plant diseases, plant growth promotion, induced resistance, and plays an important role in global sustainable agriculture. This study aimed to enhance the production of microbial xylanase in high titer from the endophytic fungus *Trichoderma harzianum* kj831197.1, and the cloning of xylanase genes in *E. coli* DH5α using a pUC19 vector. A combination of glucose, 0.1 mM, Tween 80 with lactose, and 2 mM galactose combined with malt extract boostedthe enzyme production. Xylanase production was maximized at a pH of 5.0, temp. of 30 °C, and agitation of 150 rpm in the presence of malt extract and bagasse as the best nitrogen source and waste, respectively, using submerged fermentation. The molecular weight of highly purified xylanase was 32 KDa, identified using SDS-PAGE. The xylanase gene of *T. harzianum* kj831197.1 was screened in fungal DNA using definite primers specified in the gene bank database. The identified region was excised using restriction enzymes HindIII and EcoRI and cloned into a pUC19 plasmid vector. Optimization of fermentation conditions improved xylanase production about 23.9-fold.The antifungal efficacy of xylanase toward different phytopathogenic fungi was determined. The highest inhibition was against *Corynespora cassiicola, Alternaria* sp., *Fusarium oxysporum,* and *Botrytis fabae.* This study offered an economical, simple, and efficient method using *Trichoderma harzianum* kj831197.1 for the production of the xylanase enzyme via the submerged fermentation method.

## 1. Introduction

The mandate for fungal xylanases in industrial biotechnology has displayed an unblemished intensification globally; accordingly, there is awareness of altering the circumstances of xylanases production from microbes [1]. Xylanases are an authoritative set of hydrolases that catalyze the breakage of xylopyranosyl b-1,4- linkages of xylan present in lignocellulosic materials of hardwood [2]. Endo-1,4-D-xylanases (EC. 3.2.1.8) (also known as endo-xylanases) are cell wall hydrolytic enzymes that are involved in the decomposition of xylan. Xylan is considered as a crucial part of hemicelluloses and is plentiful in monocot plants’ cell walls [3]. Endo-xylanases catalyze the hydrolysis of the -1,4 bond, which breaks down the xylan skeleton and can be valuable all over plant infections [4]. On the other hand, some cell wall-degrading enzymes (CWDEs) might have dual functions because their presence is sensed by plants, triggering defense responses. These enzymes can activate the innate immune system of plants by both pathogen-associated and damage-associated molecular patterns. Considering the commercial point of view, Xylan-degrading enzymes from varied microorganisms such as fungi, bacteria, and yeast strains have riveted extensive attention due to their prevalent application in industrial processes, viz., improvement of the digestibility of feedstocks of animals [5], pulp bio bleaching and in the textile industry [6], xylooligosaccharides production [7], enhancement of the texture in bakery products [8], juice and wine clarification, conversion of lignocellulosic wastes to appropriate reasonable products such as ethanol, sugary syrups, and liquid and gaseous fuels [9,10], animal husbandry, bread making, and fruit juice extraction [11].

Production of the xylanase enzyme by numerous organisms such as fungi [11], bacteria [12], and yeast [13,14] have been considered. Amongst microbial sources, the genus *Trichoderma* is, widely known to supply an excessive amount of xylanase enzyme, can be added to the culture medium. This property makes fungi economically powerful manufacturers of xylanases [15,16,17]. *Trichoderma* strains are non-plant pathogenic filamentous fungi that can generate an excessive yield of extracellular xylan-degrading enzymes [18,19]. *Trichoderma longibrachiatum* is the only species in this genus that is exceedingly dangerous and toxic to humans. It can create trilogies, which are poisonous peptides. As a result, a *T. longibrachiatum* infection cannot be cured with antimicrobials and severely impairs the immune system [20]. As a result, much effort has been put into improving *Trichoderma* strains to produce more industrial enzymes. Consideration should be given to enzymes produced by fungi such as *Trichoderma* sp., *T. reesei*, *T. harzianum*, and *T. viride* which seem to be good for producing xylanolytic enzymes [21]. The major products generated during the breakdown of xylan are xylooligosaccharides [21]. Its full hydrolysis is critical for obtaining higher yields of simple sugars such as D-xylose and L-arabinose, which may have implications in the fuel and food sectors [22]. *Trichoderma* sp. are frequently employed as a biological control agent in field crops to manage disease [23]. *Trichoderma* molds have been used in biotechnology as a biological control agent as well as in agriculture to combat plant infections and increase crop yields, demonstrating that this fungus can be valuable for humankind.

One of its most obvious benefits is that it eliminates competition by overcoming other fungi, making it helpful to the plants it colonizes. Some farmers like to apply it to the gardens before planting because of its numerous advantages [24,25]. This is due to the flexibility of the mechanisms of action versus agricultural diseases along with pests.

*Trichoderma* species can use several mechanisms of action such as parasitism, manufacture of antimicrobial metabolites, and manufacture of polymer- and protein-degrading enzymes (glucanases, chitinases, and proteases). Furthermore, these fungi can boost plant growth (through plant hormone production) and stimulate systemic disease resistance. Plants can also activate protective mechanisms upon contact with invaders. This is termed induced or acquired resistance [24], which makes it more applicable in agricultural settings and even more attractive [26].

The cost of the substrate has a fundamental role in the total processing fee, thus low-priced substrates as agro-residues can be used in place of carbon sources for the enzyme production process [27]. In general, xylanases are generated by two fermentation processes: submerged fermentation (SMF) and solid fermentation (SSF), which both have their own set of advantages and disadvantages. It is necessary to properly assess the worth of the predictable products, the generating microorganism, the type of the substrates, as well as the offered technical equipment when choosing one approach over the other [28]. In a previous study, approximately 80–90% of commercial xylanases were gained by SMF [29]. Some cheap agro-residues to produce xylanase are wheat bran [2], wheat husk [19], various agricultural wastes, some vegetable leaf, and groundnut shell [30,31]. Rice straw is an agricultural waste that has the potential to produce enzymes such as cellulases and xylanases, which are essential to industry [32]. Moreover, Ref. [33] reported the usage of agricultural wastes including paddy straw, coir pith, sugarcane bagasse, and leaf litter for cellulase and xylanase production. Submission of genetic engineering simplifies the large-scale production of various important industrial enzymes [34]. Genes of xylanases have been secluded from microorganisms of numerous genera and replicated in *E. coli* with the aims of enzyme production and fluctuating its properties to outfit commercial applications [35,36,37]. Therefore, this research was aimed at the production of the xylanase enzyme by *Trichoderma* *harzianum* kj831197.1 using a submerged fermentation method and a low-cost agricultural residue to establish an economical fermentation process, as well as the cloning of the xylanase gene in *E. coli* strain DH5α and the evaluation of its activity as a potent antifungal to combat the pathogenicity of a variety of plant pathogens.

## 2. Materials and Methods

### 2.1. Chemicals

Chemicals, reagents, and ingredients used throughout this study were of analytical and high-level purity grade and were obtained from Sigma (St. Louis, MO, USA).

### 2.2. Isolation of Xylanase Producing Strain from the Sugar Beet Plant

Sugar beetroots were sampled from Agricultural lands at New Borg El Arab, Alexandria, Egypt, and then washed thoroughly for ten minutes with tap water. The roots were then cut into small pieces (1 cm in diameter) and air-dried at room temperature. Surface sterilization was conducted by soaking the specimens in 76% ethanol for 1 min followed by soaking in 5.3% hypochlorite solution for 5 min and finally in 76% ethanol for 30 s. Then, they were dissected longitudinally under an aseptic condition. Soon after, they were placed on the surface of a PDA medium containing chloramphenicol (0.005% *w*/*w*). Incubation at 30 °C for 5–7 days was conducted. The fungal growth was detected, purified by streaking methods on the same isolation medium, preserved at 4 °C for further study, and sub-cultured every 21 days.

### 2.3. Identification of the Fungal Isolate

#### 2.3.1. Morphological Identification

Standard guidelines for morphological description and species identification were employed as reported by Samson et al. [38]. Colony color, reversed pigmentations, texture, and appearance were all noted down as culture features after 5 days of cultivation on potato dextrose agar (PDA). For additional characterization, the fungal isolate was examined using scanning electron microscope model JEOL-JXA-840A at the central Lab of City for Scientific Research and Technology Applications, New Borg El-Arab, 21934, Alexandria, Egypt.

#### 2.3.2. Molecular Identification

##### Genomic DNA Extraction and Primer Designing

Extraction of genomic DNA was performed using the standard CTAB technique [39]. Specific ITS primers were used to amplify DNA from the fungal isolate, i.e., ITS1: 5′-TCTGTAGGTGAACCTGCGG-3′ and ITS4: 5′-TCCTCCGCTTATTGATATGC3′ [40,41].

##### PCR and DNA Sequencing

The amplified PCR products were fractionated by running through 1.5 percent (*w*/*v*) agarose gels, which were stained with 0.5 mg mL^−1^ ethidium bromide. Estimation of the amplified fragments’ molecular weights was achieved by comparison with molecular weights of DNA markers (1 kb DNA ladder). The amplified bands (600 bp) of ITS1, 5.8 S ribosomal RNA gene; ITS2, complete sequence; and 28 S ribosomal RNA gene were excised from the gel, and the elution was refined agreeing to the producer’s guidelines using a DNA kit (Fermentas, Germany). The Applied Biosystems ABI Prism Big Dye terminator cycle sequencing kit was used to read a PCR fraction of the ITS region from the *Trichoderma* strain, using the same primers as for ITS region amplifications. A computerized sequencer (MegaBace 500) was used to depict the sequences.

### 2.4. Bacterial Strain and Culture Circumstances

*Escherichia coli* DH5α (Qiagen, Germantown, MD, USA.) was used as the replication and expression host, respectively. This strain was grown in a lactose broth medium (LB) supplemented with 50 μg/mL ampicillin (Sigma Aldrich, Burlington, MA, USA) at 37 °C.

### 2.5. Plasmids

Plasmid pUC19 (Qiagen, USA) was used as a cloning vector with a cloning site for cloning and sequencing of the xyn 2 gene.

### 2.6. Fungal Growth and Optimization of Xylanase Productivity

*T. harzianum* was grown on potato dextrose agar (PDA) at 28 °C ± 1 in the dark for 7 days. Conidiospores were gathered, washed by sterilized distilled water, and filtered using a double layer of sterile Mira cloth. The concentration of spores was determined by a Neubauer chamber. In the current study, different parameters were carried out to optimize the productivity of the xylanase enzyme. After determining each parameter, the best result obtained was applied in the subsequent parameters.

#### 2.6.1. Selection of the Best Medium Supporting Highest Enzyme Activity

Four different media were tested to find the optimal medium for maximum enzyme production. After sterilization, 1 mL of fungal spore suspension containing 4.5 × 10^3^ spores per mlwas introduced followed by incubation at 30 °C for 7 days. After the incubation period, 10,000 rpm centrifugation was conducted for 10 min. The attained supernatant was used in assessing xylanase activity.

Basal medium (M1) (g/L): (KH_2_PO_4_ 1.0 g/L, ammonium sulfate 0.5 g/L, MgSO_4_·7H_2_O 0.5 g/L, CaCl_2_ 0.01 g/L, yeast extract 0.01 g/L, CuSO_4_·5H_2_O 0.001 g/L, Fe_2_(SO_4_)_3_ 0.001 g/L, MnSO_4_·H_2_O 0.001 g/L, and glucose 20 g/L) pH 6–6.5.

Medium (M2) (g/L): KH_2_PO_4_ 1.5, NH_4_Cl_4_, MgSO_4_.7H_2_O 0.5, KCl 0.5 and yeast extract 1.0 in distilled water, 0.04 mL/L, trace element solution following component arrangement having, in µg, FeSO_4_·7H_2_O 200, ZnSO_4_·7H_2_O 180, and MnSO_4_.7H_2_O 20 at pH 6 [42].

Medium (M3) (g/L): malt extract 0.5, NH_4_SO_4_ 0.25, KH_2_PO_4_ 1.4, CaCl_2_ 2, MgSO_4_·7H_2_O 0.3, NaNO_3_ 3.0, KCl 0.5, and trace elements(mg/L): CuSO_4_·5H_2_O 0.5, FeSO_4_.7H_2_O 0.5, MnSO_4_·4H_2_O 1.6, ZnSO_4_·7H_2_O 1.4, CoCl_2_·6H_2_O 20, and 0.1% Tween 80 (*v*/*v*), pH 5.5 [43].

Medium (M4) (g/L): peptone 0.8, NH_4_NO_3_ 2.5, MgSO_4_·7H_2_O 1.5, KH_2_PO_4_ 1.2, KCl 0.6, and trace element solution of basal medium 0.3 mL/L.

In subsequent experiments, medium 3 may be used for further investigations, as it gave the highest xylanase biosynthesis. Different C-sources such as xylose and glucose were used alone and in a combination of malt extract (ME) in a conc. of 0.5% [44].

#### 2.6.2. Effect of Different pH Values

The medium used for xylanase production was adjusted at various pH values, viz., 3, 5.5, 6, and 7, using 6N NaOH and 6N HCl. One milliliter of fungal spore suspension containing 4.5 × 10^3^ CFU mL^−1^ was inoculated on the production medium and incubated under shaking conditions. Xylanase activity was assayed at the end of the incubation period.

#### 2.6.3. Effect of Incubation Conditions (Shaking and Static Manner)

This was carried out by incubating the flasks containing the inoculated production medium under shaking at different agitation rates (100, 150, or 200 rpm) while another flask was incubated under static conditions. After ending the incubation, xylanase activity was estimated.

#### 2.6.4. Effect of Different Incubation Temperature

The xylanase-producing fungal strain was allowed to grow at different incubation temperatures (25, 30, 35, 40, or 45 °C) for 7 days on a rotary shaker. At the end of the incubation interval, xylanase activity was assayed.

#### 2.6.5. Effect of Carbon Sources

Glucose, xylose, lactose, and arabinose at a concentration of 2 mM were tested as carbon sources and the assay of xylanase was conducted individually with each C source. A control lacking a carbon source was also included.

#### 2.6.6. Effect of Nitrogen Sources

Nitrogen sources’ low and high levels of each nitrogenous compound were tested for enzyme production. Urea, peptone, and yeast extracts presented at low levels as recommended in the media composition reached to its half, and high conc. presented as two times as the initial conc. presented in the original medium. Two extra N sources (beef extract and malt extract at two different conc., 0.5% and 1%) were also tested. The best nitrogen source was incorporated into the next experiment as part of the same study.

#### 2.6.7. Effect of Different Inducers

Different inducers were examined alone and in combination in this experiment, including sophorose, galactose, and mannose, all at a concentration of 2 mM.

#### 2.6.8. Effect of Tween 80 as an Additive

Concentrations of 0.1 and 0.2% *v/v* of this additive were mixed with the production medium (before autoclaving). Inoculation and incubation were carried out as previously mentioned [45,46].

#### 2.6.9. Effect of Agricultural Wastes

In this experiment, the C-N sources of the production medium were substituted with naturally occurring wastes such as rice brain, rice straw, sugarcane bagasse, wheat, and wood in three different conc. of 0.25%, 0.5%, and 1.0%. The substrates were oven-dried at 70 °C for 48 h, ground to 40 mesh particle sizes, then used as a substrate. Other previously mentioned optimal conditions were taken into consideration.

### 2.7. Purification of Crude Xylanase

Xylanase produced by *Trichoderma harzianum* isolate kj831197.1 was purified as described by [47] with minor modifications. All purification steps were conducted at 4 °C. The fractionation of the crude enzyme was carried out by (NH_4_)_2_SO_4_ at 80% saturation. Ultra filtration was managed to concentrate the enzyme. Purification of the concentrated xylanase enzyme was achieved by Sephadex G-100 columns applying 50 mM phosphate buffer, pH 7.8. Equilibration and elution of the column were achieved with the same buffer. Fractions containing xylanase were concentrated and used for further characterization.

#### 2.7.1. Polyacrylamide Gel Electrophoresis (SDS-PAGE)

The purity of the enzyme was checked using SDS-gel electrophoresis, which is also used for the determination of the molecular weight of the enzyme compared with a protein marker according to [48].The active fraction obtained was subjected to PAGE (13% polyacrylamide gel) using a vertical plate gel (Bio-Rad, Hercules, CA, USA) for detecting its molecular weight by comparing with a protein marker (in the range 6.5 to 70 kDa, Sigma).

#### 2.7.2. Characterization of Purified Xylanase

##### Substrate Specificity

Xylanase activity was determined using various substrates, viz., (xylan, laminarin, and cellulose).

##### Effect of pH

The optimum pH for purified xylanases activity was estimated by assaying enzymatic reactions in McIlvaine buffer (adapted to different pH values between 3.0 and 8.0, with one unit intervals for 140 h at 4 °C) in the privation of substrate.

##### Effect of Temperature

To determine the optimal temperature, enzymatic reactions were carried out using the purified xylanase (sodium acetate buffer 0.05M, pH5.5), applying temperatures ranging from 30 to 70 °C.

### 2.8. Bioreactor Cultivation Conditions

The bioreactor experimentations were passed out in a 3 liter bioreactor (2 liter working volume) of the previously obtained optimized medium. The bioreactor was fortified with tools and controllers for agitation, temperature, and pH. Fermentor contents were sterilized and permitted to cool, then inoculated with the preculture of fungal strain. Stirring at 150 rpm was carried out to certify homogeneous mixing, and the rate of airflow was controlled at 1 vvm while the temperature was preserved at 30 °C. The process of fermentation was continuous for 140 h. Collection of samples from the bioreactor was performed at specified intervals (6 h) for analysis.

### 2.9. DNA Manipulation

#### 2.9.1. Isolation of Xylanase Gene from *T. harzianum*

Purification of DNA directly from *T. harzianum* was achieved using a kit for genomic DNA purification (Fermentas, Germany) according to the manufacturing procedure. Oligonucleotide primers used for PCR amplification for xyn2 gene (xyn2, 1080 bp) fragment non-coding sequences were designed based on the available sequences present in the database via the GEENTOOL software. EcoR1 and HindIII restriction sites were found in the primer sequences to facilitate cloning and were obtained from Bioneer (Cheongwon, Korea). PCR amplification was carried in Eppendorf Master Cycler (Germany) using the forward oligonucleotides CKT 087 (5′-ATCGGAGTCGACACTCGCATCCG-3′; corresponding to sequences from 1075 to 1053) and the reverse oligonucleotides CKT 088 (5′-ATGCTAGCGTTGATGTCTTCT TGCTTCAGC-3′; corresponding to sequences from 1 to 23) according to [49]. Total PCR reaction volume of 25 μL was prepared, and PCR circumstances were considered. The amplified PCR product of the Xyn gene was purified using the Biospin PCR purification kit (BioerBiospin, China) and separated by electrophoresis on a 1% agarose gel. The PCR products of the xyn2 (1080 bp) gene was ligated to a pUC19 DNA vector (2.686 kb) to yield corresponding reporter plasmid (3766 bp) with inserts of the xyn2 gene and directly transformed into *E. coli* DH5α-competent cells to construct a xyn2-enriched DNA library carried out according to standard techniques [50,51,52] using the Strata cloning kit (Agilent Technology, Santa Clara, CA, USA).

#### 2.9.2. Screening of the Transformed Cells and Confirmation of Clones

The recombinant clones were identified using the blue and white colony assay. After incubation, colonies having a white color (winning recombinant vectors) were selected and splashed on the surface of Luria agar plates supplemented with Amp100, IPTG, and X-gal, then incubated overnight at 37 °C. Endorsement for the existence of anticipated fragments of DNA in the cloning vector was achieved by diverse procedures as labeled below. The alkaline lyses protocol of [53] with some changes was used for the seclusion of plasmids.

#### 2.9.3. PCR Confirmation of Recombinant PUC19 Plasmid

The PCR amplification of clones with respective primers for the xyn2 gene was used for confirming the presence of cloned fragments (3766 p). In the PCR, positive and negative controls were employed for the total DNA and cloning vector. Relative restriction examination of the indicated clones, as well as the control vector, was also used to validate the incidence of an insert with HindIII and Eco R1 for the *T*. sp. xyn2 gene (3766 bp).

#### 2.9.4. Screening of Xylanase Production

A screening test for xylanase activity of fungal xylanase-positive clones was performed by determining the amount of reducing sugars liberated using a dinitro salicylic acid (DNS) method as described by Miller [54]. One unit of the enzyme activity is defined as the amount of enzyme that liberates one µmole of xylose from xylan at 34 °C for 20 min. The colonies harboring the xylanase gene showed high enzyme activity. Recombinant DNAs were isolated from these clones and analyzed with EcoR1 and HindIII restriction enzymes to determine the size of the inserts.

#### 2.9.5. Extraction of Xylanase Gene and Assay of Enzyme

To evaluate the localization of the recombinant xylanase in *E. coli* cells, a suitable amount of the fermentation medium was spun (10,000× *g* for 10 min at 4 °C). Next, the supernatant was utilized to assess xylanase activity. Then, 0.5 mL of the supernatant containing the enzyme was mixed with0.5 mL of the substrate solution (birchwood xylan) (tubes incubated at 34 °C in a water bath for 20 min, stirred gently by hand from time to time. Finally, a DNS reagent was added to halt the reaction. The tubes were boiled for 10 min at 100 °C. The blank tubes were prepared similar to sample tubes, only exchanging enzyme supernatant with distilled water. Spectroscopic absorbance was measured at 400 nm against the blank. One unit of the enzyme activity was defined as the amount of enzyme that liberates one µmole of xylose from xylan at 34 °C for 20 min. The amount of reducing sugars was quantified spectrophotometrically at 400 nm using 3.5 dinitro salicylic acid (DNS).A procedure utilizing xylose solution was the reference standard. All assays were accomplished in triplicate, and the results are described as mean ± standard deviation.

#### 2.9.6. Antifungal Activity Assay

The fungal strains *Alternaria alternate* isolate STE-U4349, *Botrytis fabae* isolate MU BF1, *Fusarium oxysporum* isolate KJ831189, *Fusarium solani* isolate KJ831188, *Fusarium avenaceum* isolate P21 70, *Corynespora cassiicola* isolate YC46, *Fusarium decemcellulare* isolate C82NL, and *Bipolaris oryzae* isolate 232 were obtained kindly from the Plant Pathology lab, Arid Lands Cultivation Research Institute, Borg El Arab, Alexandria, Egypt.

Antifungal efficacy of crude xylanase was assayed using the hyphen extension inhibition assay, as described by [55], with a few variations. Fungal mycelium was inoculated at one pole of a (PDA) Petri plate which was then incubated at 28 °C for 24 to 48 h. Next, 200 μL of the xylanase enzyme under study was spotted in the front of the growing fungal mycelium. Finally, additional incubation of plates at 28 °C was performed for 7 days and the progress of mycelia inhibition was recorded. The antifungal activity was measured in terms of percentage of mycelia inhibition according to the following equation:

Inhibition Percentage = [(d _control_ − d _treatment_)/d _control_] × 100.

### 2.10. Statistical Analysis

The data analysis was achieved using computerized software SPSS (Statistical Package of Social Science, version 20, IBM Corp, Armonk, NY, USA). Duncan’s multiple comparisons test was employed to assess the variance between the investigational groups, and *p* < 0.05 was chosen as the statistical significance level.

## 3. Results

The main objective of the present studywas to explore the screening, production, and optimization of xylanase enzymes from *Trichoderma harzianum* kj831197.1 with the aid of the submerged fermentation method for establishment of an economical fermentation process and cloning of the xylanase gene in *E. Coli* strain DH5α.

**Figure 1:**
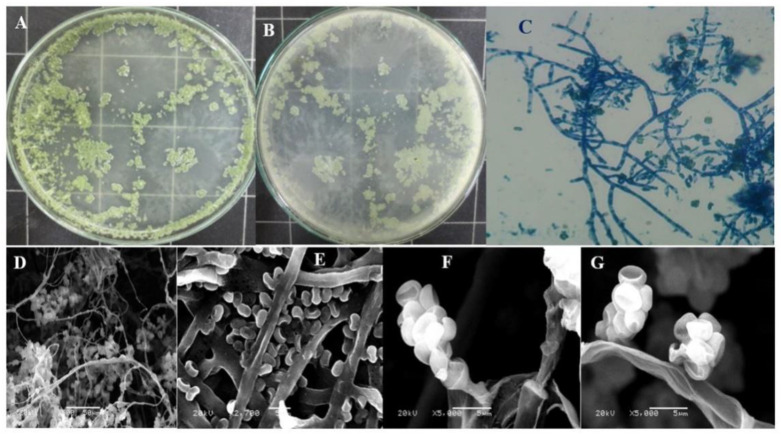
Culture characteristics ((**A**) = front and (**B**) = reverse) of *Trichoderma harzianum* kj831197.1 on PDA media after 5 days of incubation, (**C**): microscopic features of fungal mycelia and spore under the light microscope (40×), and (**D**–**G**): different internal structures of conidia and conidiophore under the scanning electron microscope (500, 2700, and 5000 magnification), respectively.

A total of five fungal isolates were obtained from sugar beet roots. Only one isolate was selected according to its ability to produce the xylanase enzyme, based on our previous preliminary results using the quantitative method for assaying enzyme activity (Figure 1).

### 3.1. Phylogenetic Relationships

The achieved *T. harzianum* sequence was saved in the gene bank with accession number (KJ831197.1). The strain showed a 98.1% homology range. A dendrogram based on the ITS region partial sequences of several species and strains of the genus *T. harzianum* were depicted by neighbor-joining analysis of the NJ method and the maximum composite likelihood method.

**Figure 2:**
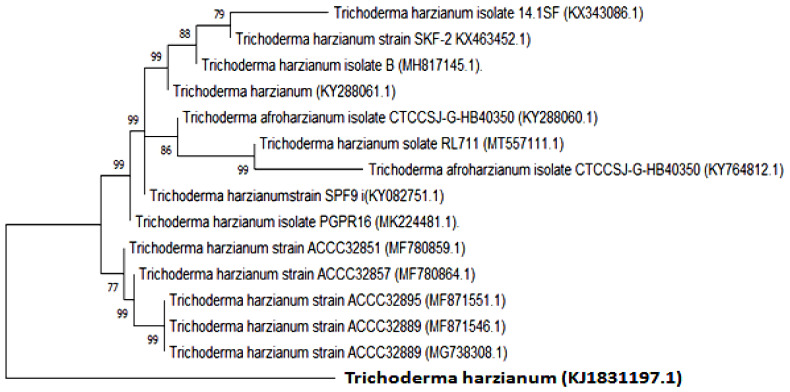
The phylogenetic tree shows the phyletic relationships among the *T. harzianum* (KJ831197.1) isolate and other eight matching homologous species and strains from the NCBI gene bank database. The DNA substitution model was the p-distance (nucleotide)method, and the phylogenetic tree construction was depicted by the construct and test neighbor-joining tree model according to Saitou N. and Nei’s (1987) NJ method.

The obtained sequence was used to extract the most closely related sequences among *T. harzianum* using the BLASTn search. Alignments were produced for phylogenetic analyses using ClustalX 2.1.0.12 software 9 [56] with default parameters. The phylogenetic tree was constructed with maximum likelihood (ML) and neighbor-joining (NJ) methods using MEGA software, version 10.1.6 [57]. The optimal tree with the sum of branch length = 0.00733945 is shown. The percentage of replicate trees in which the associated taxa clustered together in the bootstrap test (1000 replicates) is shown next to the branches. The evolutionary distances were computed using the p-distance method (Figure 2).

### 3.2. Optimization of Xylanase Productivity

**Figure 3:**
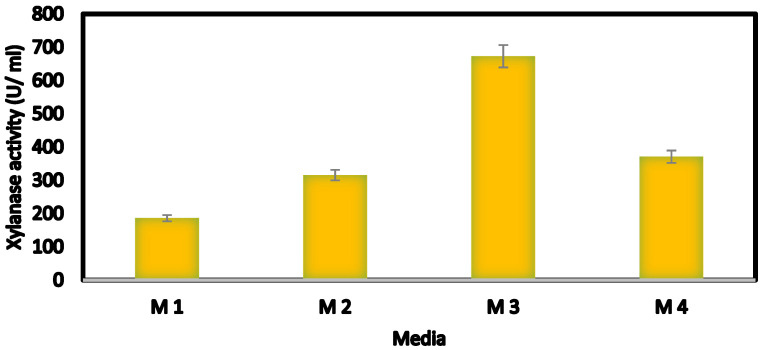
Effect of different culture media on xylanase productivity by *Trichodermaharzianum* kj831197.1.NB:M1: basal medium, M2: Paul et al. (1999), M3: Mandels and Sternburg (1976), and M4: Haltrich et al. (1994).

A screening test for xylanase productivity using different medium types resulted in the fact that medium no. 3 was the best medium for maximal production of xylanase as illustrated in Figure 3.

**Figure 4:**
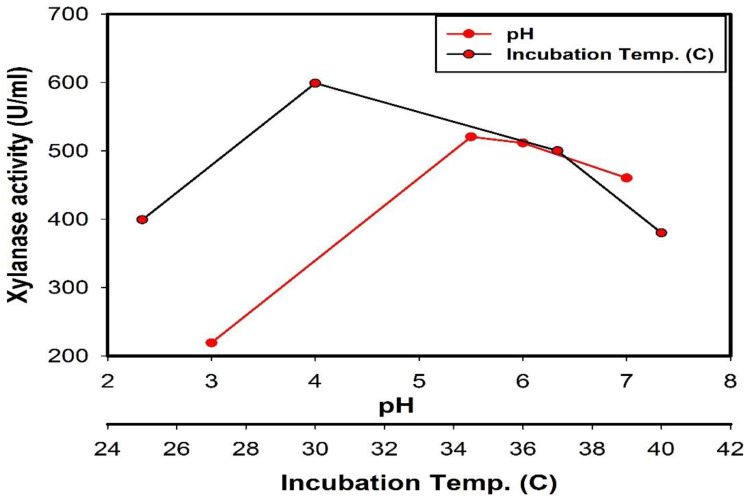
Relation of different incubation temperatures (°C) and pH values to xylanase production by *Trichoderma harzianum* kj831197.1.

The impact of changing the medium pH from 3 to 7 on xylanase production was observed. On the clearest data, the highest xylanase productivity was observed at pH 5.5 (520.56) as shown in Figure 4.

Commonly, microbes are acknowledged to yield high levels of enzymes at their optimal growth temperature. In the current study, 30 °C was the optimum temperature for maximal production of xylanase by *Trichoderma harzianum* kj831197.1 (Figure 4).

**Figure 5:**
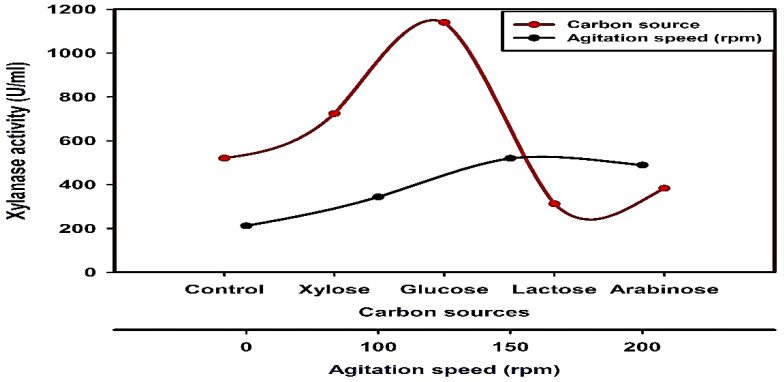
Comparing the effect of using different carbon sources and agitation speeds on xylanase production by *Trichoderma harzianum* kj831197.1.

Glucose was observed to exhibit the highest xylanase production when compared with xylose, lactose, and arabinose (Figure 5). To gain a more detailed understanding of the production of xylanase by *Trichoderma harzianum* kj831197.1, different incubation conditions such as static and shaking were carried out. Results in Figure 5 showed that shaking conditions proved to be more preferable for the production of the xylanase enzyme than static conditions by *Trichoderma harzianum* kj831197.1, especially at 150 rpm.

**Figure 6:**
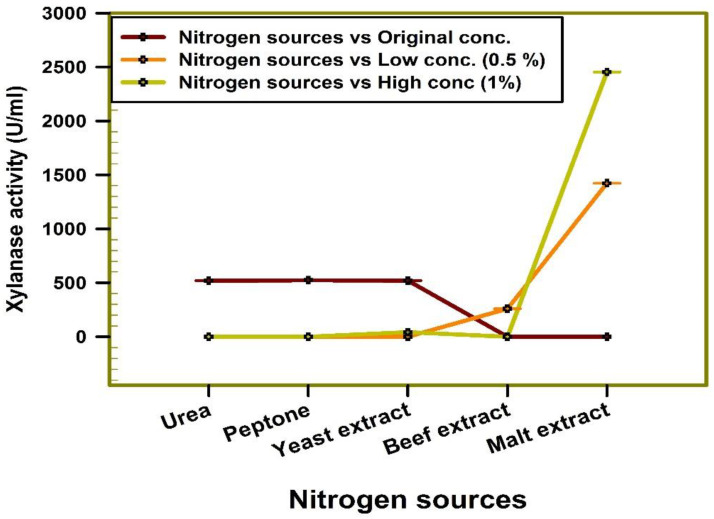
Relation of different nitrogen sources to xylanase production by *Trichoderma harzianum* kj831197.1.

The results gained throughout the study suggest that malt extract was the most suitable nitrogen source as it offered the maximal activity of enzyme under study as represented in Figure 6. The experimentation yielded a satisfactory outcome, five-fold greater in xylanase activity, equated with the non-optimized basal medium.

The impact of distinct concentrations of Tween 80 on enzyme production was studied. The addition of Tween 80 to the medium improved the production of the xylanase enzyme. Two concentrations of Tween 80 (0.1 mM and 0.2 mM) were used in the presence of carbon source xylose and lactose compared with basal media where glucose was used as the C-source. The results showed that high enzyme activity (2192.68 U/mL) was obtained from low conc. of Tween 80 with lactose and decreased by increasing Tween conc. as shown in Table 1. The initiation of xylanase was strongly reliant on the concentration of Tween 80.

Results for inducers’ impact on xylanase production are given in Table 2. By adopting the mixture of galactose and malt extract, the xylanase activity reached (2636 U/mL) when glucose was added as a carbon source.

Five agricultural wastes were used to investigate the most suitable substrate for xylanase production from *Trichoderma harzianum* kj831197.1. These wastes were wheat bran, sugarcane bagasse, rice bran, rice straw, and wood (Table 3). The highest yield of xylanase enzyme production (3620.19 U/mL and 2910 U/mL)was obtained when sugarcane bagasse and wood were used as substrates, respectively.

From the obtained results, the enzyme production under the optimum conditions was increased 6.5-fold compared with the amount obtained at the first screening production.

### 3.3. Purification of Xylanase Enzyme

The properties of xylanase from *Trichoderma harzianum* kj831197.1 were judged by using ammonium sulfate precipitation and Sephadex G-100 (Table 4).In a trial to precipitate xylanase by ammonium sulfate, the results revealed that increasing the concentration of ammonium sulfate gave rise to an increase in the specific activity of xylanase (up to 80% saturation).Gel filtration of xylanase on Sephadex G-100 showed that there was one active peak (from fraction15 to 17) in which fraction number 16 reached the highest specific activity up to (433 U/mg).The fold purification of xylanase was 23.9.

### 3.4. Characterization of Purified Xylanase

#### 3.4.1. Substrate Specificity

**Figure 7:**
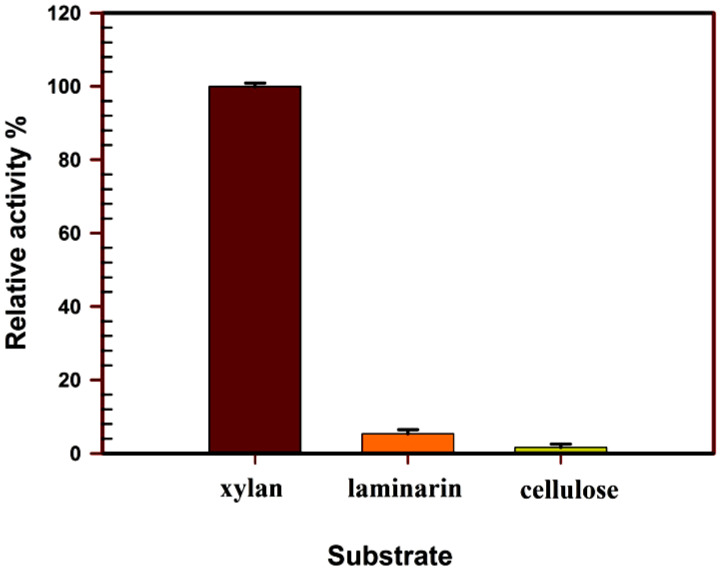
Substrate specificity of purified xylanase.

The purified xylanase from *Trichoderma harzianum* kj831197.1 showed the highest substrate specificity toward xylan with 100% relative activity, while having low activity toward cellulose and laminarin (Figure 7).

#### 3.4.2. Temperature and pH Stability

**Figure 8:**
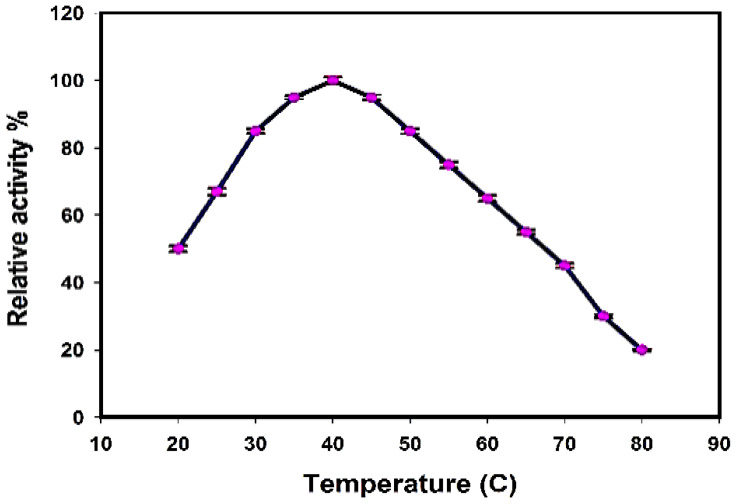
Temperature stability of purified xylanase.

**Figure 9:**
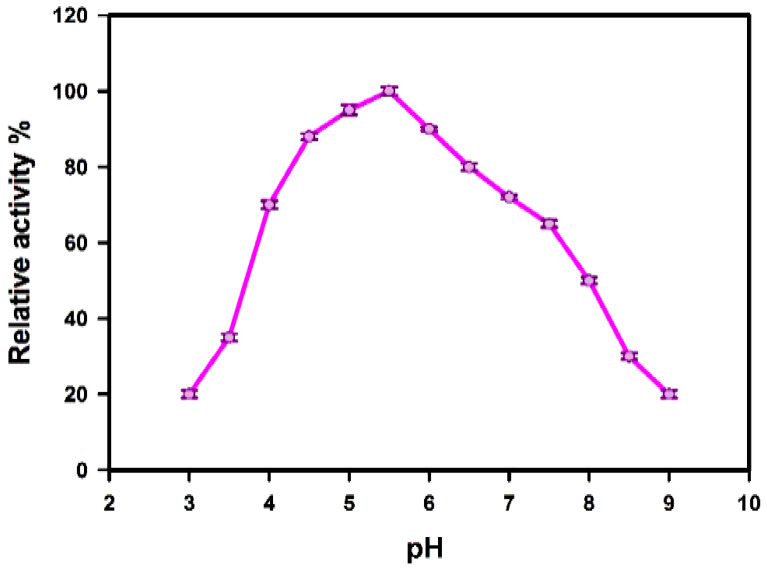
pH stability of purified xylanase.

The enzyme activities concerning temperature and pH stability were studied. The results of the purified enzyme indicated that the optimum temperature and pH for the xylanase enzyme were at 40 °C and 5.5, respectively (Figure 8 and Figure 9).

#### 3.4.3. Polyacrylamide Gel Electrophoresis (SDS-PAGE)

**Figure 10:**
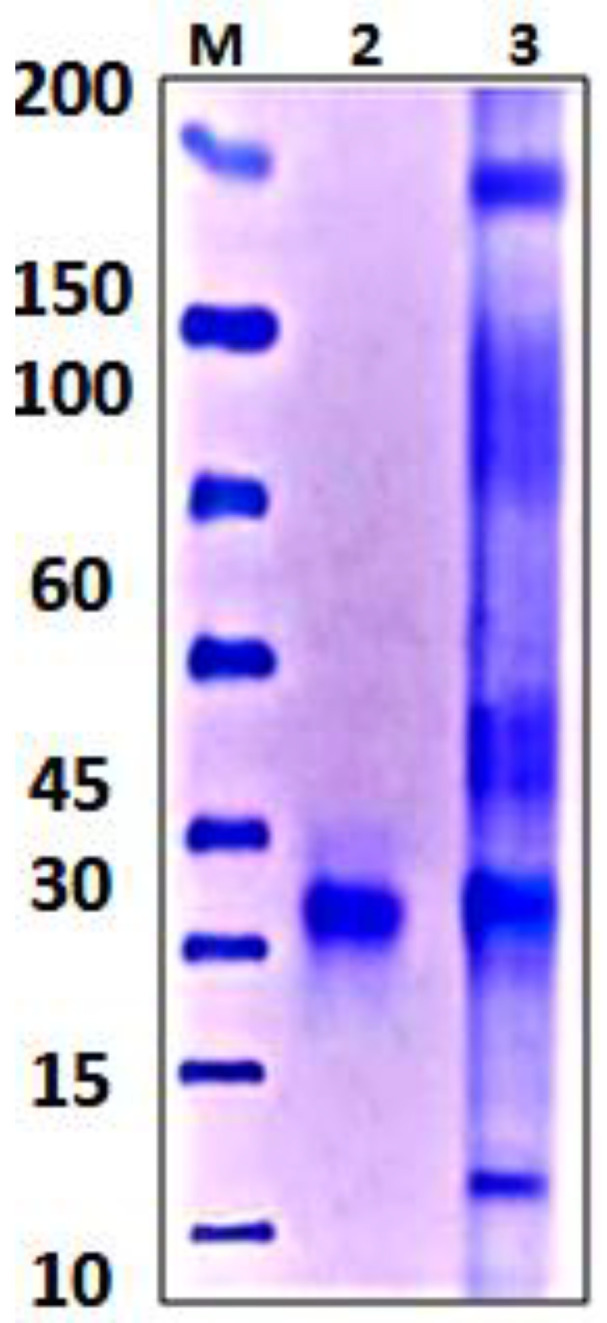
SDS-PAGE of purified xylanase; Lane (M): marker, Lane2: purified xylanase, and Lane 3: crude xylanase.(Protein Ladder, ^®^Extra broad, molecular weight 6.5–270 KDa, Abcam, Waltham, MA, USA).

The purified xylanase enzyme produced by *Trichoderma harzianum* kj831197.1. was subjected to SDS-PAGE analysis. The molecular weight of the pure xylanase was estimated at 32 KDa. Figure 10 indicates that the purified xylanase produced by *T. harzianum* kj831197.1contained a single protein band.

### 3.5. Cultivation Conditions for Large-Scale Production of Xylanase Enzyme in a Bioreactor

**Figure 11:**
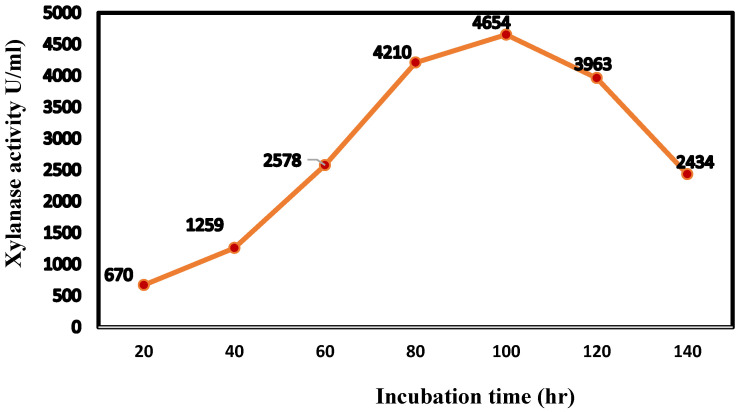
Xylanase activity profile in the bioreactor.

During research in our lab, it was noticed that the speed of agitation, which sprays certain fragments of mycelia on the wall vessel, amplified the mycelium development of the fungi during culture performance. The behavior of the selected strain *Trichoderma harzianum* kj831197.1 in the laboratory by the bioreactor was analyzed, allowing continuous tracking of the fermentation process data (temperature, pH, and dissolved oxygen concentration). The greatest xylanase activity was 4654 U/mL, which was 6.5 times higher than the original medium and achieved in a shorter period (almost half the time taken in the shaking flask) (Figure 11). Galactose is thought to improve respiration, cell viability, and energy consumption by allowing oxygen to enter the cells more easily. An industrial-scale bioreactor should be constructed and regulated to produce the highest amount of product in the shortest length of time with the lowest cost.

### 3.6. Isolation and Transformation of Xylanase Gene

The xylanase gene was isolated by PCR amplification using chromosomal DNA from *Trichoderma harzianum* kj831197.1 as a template. The PCR product of amplified genes was digested with the HindIII/EcoRI and cloned into the same sites in the pUC19 (2.686 kb) plasmid and transformed into *E. coli* DH5α cell.

#### 3.6.1. Screening of the Transformed Cells

The confident colonies that developed on LB plates supplemented with Kanamycin were exposed to PCR using primers of the xylanase gene and the PCR circumstances that were previously cited in the materials and methods section for an additional assortment of colonies sheltering plasmid with the xylanase gene. The recombinant clones were detected with the aid of using the blue and white colony assay. Subsequent to incubation with white colonies only, recombinant vectors were selected and applied on the surface of Luria agar plates supplemented by Amp100, X-gal, and IPTG. The incubation of plates was applied all night at 37 °C.

#### 3.6.2. Confirmation Using Restriction Analysis

**Figure 12:**
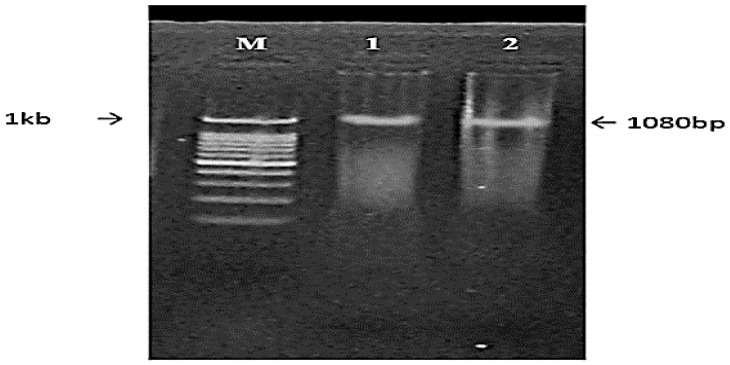
Agarose gel electrophoresis of PCR amplification of xyn2 gene (1080 bp). M, 3 kb DNA marker; Lane 1, xyn2 gene; Lane 1, 2.

**Figure 13:**
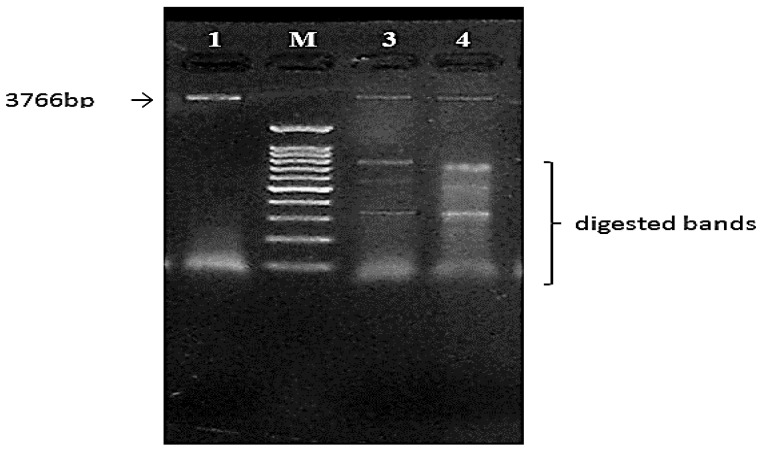
Agarose gel electrophoresis of restriction analysis of pUC 19 clones with inserted xylanase 2 genes digested by HindIII and EcoR1, control pUC19 (3766 bp); Lane 1, M, 1.5 kb DNA marker; Lanes 3 and 4, recombinant Xyn2 genes digested DNA digested with EcoRI and HindIII, respectively.

To see if the plasmid included the xylanase fragment, HindIII and Eco R1 were used to digest selected clones and the control vector (recombinant and non-recombinant plasmids) for the *Trichoderma harzianum* kj831197.1 xyn gene. The obtained results existing in Figure 12 showed that one fragment of roughly 3766 bp was produced by the recombinant plasmid compared to the 1 kb DNA marker, and these findings indicated that the recombinant xylanase gene was efficaciously expressed in the *E. coli* DH5α host cell (Figure 13).

#### 3.6.3. Estimation of Xylanase Activity

The activity of the xylanase gene from stimulated and non-stimulated mutated *E. coli* was assessed. The overall xylanase activity was9 U/mL, which was lower than the activity found in *Trichoderma harzianum* kj831197.1 (80 U/mL).

#### 3.6.4. In Vitro Antifungal Activity of Crude Xylanase

**Figure 14:**
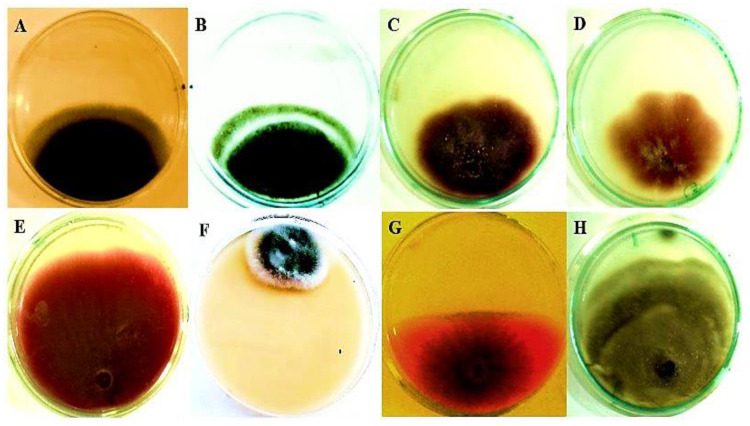
Antifungal activity of crude xylanase against some plant pathogenic fungi (**A**) *Alternaria alternate* STE-U4349, (**B**) *Botrytis fabae* MU BF1,(**C**) *Fusarium oxysporum* KJ831189, (**D**) *Fusarium solani* KJ831188, (**E**) *Fusarium avenaceum* P21 70, *(***F**) *Corynesporacassiicola* YC46, (**G**) *Fusarium decemcellulare* C82NL, and (**H**) *Bipolaris oryzae* 232.

The effect of *Trichoderma harzianum* kj831197.1 xylanase on the growth of some plant pathogenic fungi isillustrated in Table 5 and Figure 14. Results showed thatbetter antifungal action and the highest inhibition was against *Corynespora cassiicola* YC46, *Alternaria alternate* STE-U4349, *Fusarium oxysporum* KJ831189, *Botrytis fabae* MU BF1, *Fusarium decemcellulare* C82NL, and *Fusarium solan* iKJ831188(81, 63, 60, 58, 56, and 54%), and the feeblest effect was on *Fusarium avenaceum* P21 70 and *Bipolaris oryzae* 232 (20%) for each.

## 4. Discussion

In a preliminary study, we screened the beet sugar plant endophytic fungus for its activity in producing the xylanase enzyme. The potential xylanolytic-producing fungus identified as *Trichoderma harzianum* kj831197.1 is a member of the family Hypocreaceae. *Trichoderma* strains are non-plant-pathogenic filamentous fungi that can generate an excessive yield of extracellular xylan-degrading enzymes [18,19].

By screening different medium types for xylanase productivity, it was found that medium no. 3 was the best medium for maximal production of xylanase. It is widely known that 30 to 40% of the manufacturing price of commercial enzymes is taken up with the cost of the growth medium [58]. Moreover, some other workers reported that wastewater resulting from the pulp industry could be used as a medium for the production of xylanase [59].

The highest xylanase productivity was observed at a pH of 5.5 and a temperature of 30°C. The hydrogen ion concentration of the medium is one of the most monitored parameters during the fermentation process [60]. It was reported that the pH of the medium can affect enzyme production since it can affect both solubility and ionization of medium substrates and their availability for fungal growth [61]. The effect of altering the medium pH from 3 to 7 has been observed on xylanase productivity. On the clearest data, the highest xylanase productivity was observed at a pH of 5.5 (520.56). Parichart et al. [62] recorded that the ideal pH for xylanase productivity from *Aspergillus niger* was 6.0 while Sonika et al. [63] denoted that a pH of 6.0 gave the maximum xylanase activity from *Trichoderma* sp. Generally, pH values around 5.0 have been informed to be optimal for xylanase production [64,65]. Fungi generally prefer slightly acidic conditions.

Commonly, microbes are acknowledged to yield high levels of enzymes at their optimal growth temperature. Long et al. [16] informed that the optimal temperature intended for xylanase enzyme production from *Trichoderma orientalis* was 37.3 °C while the ideal temperature for xylanase enzyme invention from *Trichoderma* sp. was 30 °C [63]. Moreover, some investigators showed that the optimal temperature for xylanase production under solid-state fermentation conditions from *Trichoderma* sp. was 55 °C [63,64,65,66,67]. Lower and higher temperatures reduce the specific activities for the reason that the thermal effects of such temperatures on both the growth of microorganisms and the rate of enzymatic reaction inside the cells imitate the vital construction of the enzyme [68,69].

In the current study, glucose exhibited the highest xylanase production. In contrast, xylanase activity was induced by xylose and xylan in *A. pullulans* Y-2311-1 [70]. Additionally, malt extract was the most suitable nitrogen source as it offered the maximal activity of the enzyme under study. A study conducted by [63] indicated that corn powder was the superlative nitrogen source that furnished the maximal xylanase activity by *Trichoderma* sp. under solid-state fermentation conditions. Ref. [71] reported that peptone in combination with yeast extract exhibited the uppermost xylanase activity by *Bacillus pumilus* SV-85 S. Enhancing enzyme production by Tween 80 might be correlated with the increased cell membrane permeability, permitting a more rapid secretion of the enzymes, which leads to greater enzyme synthesis as denoted by [72,73]. An additional conceivable explanation is that Tween 80 affects the level of glycosylation and consequently, protein stability [74].

The highest yield of xylanase enzyme production (3620.19 U/mL and 2910 U/mL) was obtained when sugarcane bagasse and wood were used as substrates, respectively. Because sugar cane bagasse is a biomass resource that is abundant year after year, this strategy can be utilized to add value to it by producing lignocellulolytic enzymes including cellulase and xylanase. A study conducted by [75] showed that the superlative medium for the production of xylanase from *A. niger* contains wheat bran and maltose as a carbon source. Moreover, rice straw was the most promising substrate for the production of xylanase by *Aspergillus oryzae* MN894021 [17]. Usage of wheat straw and wheat bran as a promising carbon source for xylanase production also has been described by [76,77]. Other agro-residues such as corn cob [78,79], oat spelled xylan [80], and brewer’s spent grain [81] have also been conveyed as an appropriate substrate for xylanase production. As a result, the use of these wastes in enzyme production minimized the cost of production and overcame environmental pollutants. By comparing the amount of the enzymes with the initial production from basal media, there was a notable increase in xylanase production up to 4.5 times by using these wastes. These wastes are generally recyclable and can be converted into valuable products which include biofuels, chemicals, and low-cost sources of energy for fermentation, as well as improved livestock feed and human nutrition as documented by [82].

In this study, after 140 h of cultivation of *Trichoderma harzianum* kj831197.1 in the bioreactor, the examination of the dynamic growth revealed the absence of a lag period, a steep exponential phase descent, a stationary phase with a downward appearance, and a decreasing phase. Due to differences in biotechnological factors, the aspects for growing curves in the bioreactor were higher than that achieved in conical vessels through stirring, namely: agitation circumstances and increasing volume of aerated media. The simulation findings demonstrated the systems’ control capability, which was achieved by estimating the substrate nourishment required for the development of cells as the biomass concentration increased. Biomass in a bioreactor reached a maximum after 100 h of cultivation, which was similar to the value predicted by simulation. In the case of xylanase concentration, dissolved oxygen consumption remained constant throughout the culture period. The highest biosynthesis was attained throughout the exponential growth stage, once xylose was employed as a source of carbon and especially following the induction by galactose, for the strain *Trichoderma harzianum* kj831197.1, which offers energetic growth through xylanase productivity. After 100 h of cultivation, the xylanase production curve revealed a strong peak, indicating that when biosynthesis reached its maximum, it was feasible to create a complex of enzymes. The catabolic suppression was lessened as substrate consumption increased, and microbial cells resumed biogenesis, as evidenced by the graph’s second maximal. The drop-in activities beyond 100 h of incubation were clarified by a decline in the cell population during the decline phase and catabolic suppression followed by enzyme biosynthesis throughout the process.

In our study, the xylanase gene of *T. harzianum* kj831197.1 was isolated from DNA via definite primers specified in the gene bank database, digested with HindIII and EcoRI, and cloned into a pUC19 plasmid vector. According to some researchers, recombinant xylanase productivity was less than that of the mother cell because of its ease of manipulation, suitable growing conditions, minimal transformation requirements, and ability to collect significant amounts of productivity in the cell cytoplasm. Even though *E. coli* is a reliable replicating host for recombinant proteins, successful and efficient expression of various xylanase genes is not feasible with this bacterium. This could be owing to uncommon codons appearing frequently and requiring particular translational adjustments (disulfide bond formation and glycosylation) [83,84,85].

The antagonistic reactions of xylanase toward different phytopathogenic fungi had a significant part in biocontrol and the highest inhibition was against *Corynespora cassiicola, Alternaria* sp., *Fusarium oxysporum*, and *Botrytis fabae.* Our findings agreed with [17] who stated that *Aspergillus oryzae* MN894021 xylanase T1 had significant antifungal activity and is a practical and reliable enzyme showing potential as a plant pathogen management alternative to standard chemical fungicides. Moreover, Kumar et al. [86] revealed the promising plant’s beneficial impact on *Trichoderma* sp. in suppressing *Fusarium* wilt disease in tomatoes. *Trichoderma’s* ability to create cell wall-degrading enzymes such as cellulase and xylanase seem to play a significant role in antagonistic effect [87]. Several researchers have discovered that numerous *Trichoderma* species are capable of preventing *Fusarium* wilt in a variety of agriculture crops [88]. *Trichoderma’s* mode of action varies depending on the pathogen. Their behavior differs according to the species [89]. During *Trichoderma’s* antagonistic activity, chitinase, xylanase, and glucanase are important enzymes in the disintegration of plant pathogenic fungal cell walls [90].

In a trial to precipitate xylanase by ammonium sulfate, the results revealed that increasing the concentration of ammonium sulfate increased the specific activity of xylanase up to 80% saturation.

Following the present results, Bagewadi et al. [47] recorded that 80% ammonium sulfate saturation was the best for xylanase purification from *Penicillium citrinum* isolate HZN13.

The purified xylanase and standard enzyme were subjected to SDS-PAG electrophoresis. The produced gel indicated that purified xylanase from *Trichoderma harzianum* kj831197.1 was separated into one single band of a molecular weight of approximately 32 KDa. In the findings of other studies, Ujiie et al. [91] recorded that the purified *T. viride* xylanase was assessed to be 22 KDa, while that from *T. harzianum* was estimated at 20 KDa as stated by Tan et al. [92].

The enzyme activities concerning temperature, pH, and substrate specificity were studied. The results of the purified enzyme indicated that the optimum temperature and pH for the xylanase enzyme were at 40 °C and 5.5, respectively. Fairly, the pH and temperature optima considered for the *Trichoderma harzianum* kj831197.1 xylanase in this study were consistent with those reported from the *Trichoderma* strains that were mostly observed in the pH range from 3.5 to 6.0 and in temperatures extending from 40 °C to 60 °C [19].

## 5. Conclusions

In this study, gene encoding xylanase was cloned in a suitable host to maximize the enzyme production by adjusting nutritional and environmental parameters and changing its characteristics to suit valuable purposes as a plant pathogenic biocontrol agent. This assisted in the resolution of problems such as enzyme availability, substrate scope, and operational stability. This could provide information on the present state of cloning and expression of fungal xylanases and their potential applications. Our results indicated that the optimum conditions for xylanase production were massively prompted by glucose, 0.1 mM Tween 80 with lactose, and 2 mM galactose combined with malt extract as an inducer. The maximum xylanase production was attained at a pH of 5.0, temp. of 30 °C, and agitation of 150 rpm in the presence of malt extract and bagasse as the best nitrogen source and waste, respectively, using the submerged fermentation method. Moreover, superior antifungal action was obtained by the xylanase enzyme under study and the highest inhibition was against *Corynespora cassiicola*, *Alternaria* sp., and *Fusarium oxysporum*. The recombinant clone was expressed and excreted in culture supernatant with xylose acting as an inducer. The xyn2 gene from *T. harzianum* kj831197.1 generated the largest (23.9-fold) xylanase activity compared to the wild type. The molecular weight of highly purified xylanase was 32 KDa with SDS-PAGE. The purified enzyme showed the highest substrate specificity toward xylan. This study opened the door to an opportunity of examining different fungal hosts able to form recombinant xylanases. Further technical improvements in the growth and progress of fungal expression systems by genetic engineering techniques can aid in the hyper-expression of xylanases for industrial and agricultural applications.

## Figures and Tables

**Table 1:** Relation of Tween 80 concentrations to xylanase production by *Trichoderma harzianum* kj831197.1.

Tween 80 Conc. (mM)	Xylanase Activity (U/mL)
0.1 mM + xylose	1139.9 ± 0.02
0.1 mM + lactose	2192.7 ± 0.9
0.2 mM + xylose	347.68 ± 0.5
0.2 mM + lactose	102.58 ± 0.44

All results are reported as the mean ± standard error (significance *p* < 0.05).

**Table 2:** Theeffect of different inducers on the production of xylanase by *Trichoderma harzianum* kj831197.1.

Inducer	Xylanase Activity (U/mL)
Galactose	850 ± 0.3
Sophorose	712 ± 0.9
Mannose	590 ± 0.9
Galactose + malt extract	2636 ± 0.7
Sophorose + malt extract	2150 ± 1.1
Mannose + malt extract	1430 ± 1.4

All results are reported as the mean ± standard error (significance *p* < 0.05).

**Table 3:** The consequence of using some agricultural wastes on xylanase activity by *Trichoderma harzianum* kj831197.1.

Agricultural Waste Concentration (%)	Xylanase Activity (U/mL)				
	Different Agricultural Wastes				
	Rice Bran	Rice Straw	Sugarcane Bagasse	Wheat Bran	Wood
0.25	1220.5 ± 0.72	843.9 ± 0.35	1250 ± 0.56	200 ± 0.1	2910 ± 0.40
0.5	3100.1 ± 0.48	2550 ± 0.12	2800.2 ± 0.82	319 ± 0.46	2800 ± 0.85
1	850.2 ± 0.30	2170.3 ± 0.40	3620.2 ± 1.25	510 ± 0.32	1420.1 ± 0.66

All results are reported as the mean ± standard error (significance *p* < 0.05).

**Table 4:** Purification summary of xylanase from *Trichoderma harzianum* kj831197.1.

Step	Total Activity (U)	Total Protein (mg)	Specific Activity (U/mg)	Purity (Fold)
**Crude xylanase**	105	580	18.10	1
**Ammonium sulfate precipitation (80%)**	58	107	54.2	2.99
**Sephadex G-100**	39	9	433	23.9

**Table 5:** Antifungal activity of crude xylanase against some plant pathogenic fungi.

Fungal Strain	Inhibition %
***Alternariaalternate*** STE-U4349	63
***Botrytis fabae*** MU BF1	58
***Fusarium oxysporum*** KJ831189	60
***Fusarium solani*** KJ831188	54
***Fusarium avenaceum*** P21 70	20
***Corynespora cassiicola*** YC46	81
***Fusarium decemcellulare*** C82NL	56
***Bipolaris oryzae*** 232	20

## Data Availability

This article provides all of the data collected or analyzed during this study.
